# Modern Iron Ooids of Hydrothermal Origin as a Proxy for Ancient Deposits

**DOI:** 10.1038/s41598-019-43181-y

**Published:** 2019-05-08

**Authors:** Marcella Di Bella, Giuseppe Sabatino, Simona Quartieri, Annalisa Ferretti, Barbara Cavalazzi, Roberto Barbieri, Frédéric Foucher, Fabio Messori, Francesco Italiano

**Affiliations:** 1 Istituto Nazionale di Geofisica e Vulcanologia (INGV), Sezione di Palermo, Via Ugo La Malfa 153, 90146 Palermo, Italy; 2 Dipartimento di Scienze Matematiche e Informatiche, Scienze Fisiche e Scienze della Terra (MIFT), Università di Messina, Viale Ferdinando Stagno d’Alcontres 31, 98166S Agata, Messina Italy; 3 Dipartimento di Scienze Chimiche e Geologiche (DSCG), Università di Modena e Reggio Emilia, Via Campi 103, 41125 Modena, Italy; 4 Dipartimento di Scienze Biologiche, Geologiche e Ambientali (BiGeA), Università di Bologna, Via Zamboni 67, 40126 Bologna, Italy; 5 Department of Geology, University of Johannesburg, PO Box 524 Auckland Park, 2006 Johannesburg, South Africa; 6 Centre de Biophysique Moléculaire (CBM), Rue Charles Sadron, 45071 Orléans, Cedex 2 France; 7 Department of Earth Sciences, University of Geneva, Rue des Maraîchers 13, 1205 Geneva, Switzerland

**Keywords:** Geochemistry, Sedimentology, Volcanology

## Abstract

We constrained the origin and genetic environment of modern iron ooids (sand-sized grains with a core and external cortex of concentric laminae) providing new tools for the interpretation of their fossil counterparts as well as the analogous particles discovered on Mars. Here, we report an exceptional, unique finding of a still active deposit of submillimetric iron ooids, under formation at the seabed at a depth of 80 m over an area characterized by intense hydrothermal activity off Panarea, a volcanic island north of Sicily (Italy). An integrated analysis, carried out by X-ray Powder Diffraction, Environmental Scanning Electron Microscopy, X-ray Fluorescence and Raman spectroscopy reveals that Panarea ooids are deposited at the seafloor as concentric laminae of primary goethite around existing nuclei. The process is rapid, and driven by hydrothermal fluids as iron source. A sub-spherical, laminated structure resulted from constant agitation and by degassing of CO_2_-dominated fluids through seafloor sediments. Our investigations point the hydrothermal processes as responsible for the generation of the Panarea ooids, which are neither diagenetic nor reworked. The presence of ooids at the seawater-sediments interface, in fact, highlights how their development and growth is still ongoing. The proposed results show a new process responsible for ooids formation and gain a new insight into the genesis of iron ooids deposits that are distributed at global scale in both modern and past sediments.

## Introduction

Calcareous ooids are common components of both modern and ancient sediments^1^. On the contrary, iron ooids constitute a characteristic Time-Specific Facies well documented in the fossil record since Precambrian times^2^, but getting extremely rare in modern settings. The origin and genesis of fossil iron ooids and oolitic ironstones have long been a matter of debate and controversy^1,3–9^ (did they form in shallow or deep-water, marine *vs* non-marine; e.g.^10^), and both abiotic^11^ and biologically induced^12,13^ formation mechanisms have been advanced.

Sorby^14^ first proposed the replacement of calcareous ooids as a possible genetic process. Volcanic ash was suggested to be the source for the Upper Devonian iron-replaced calcareous ooids in Belgium^15^. Kimberley^3,16^ suggested that ooidal ironstones from Cape Mala Pascua (Venezuela) were generated by the large-scale replacement of ooidal limestones by iron-rich pore waters. More recently, the contribution of iron-oxidizing bacteria and/or fungi has also been indicated^17^.

The absence of modern analogues has, however, hampered a satisfactory and unequivocal model for the origin of iron ooids. The most recent *in situ* iron ooid deposit (4,500 ka) described to date is represented by the shallow-marine volcanic material from Mahengetang, Indonesia^4^. A combined mineralogical-chemical investigation revealed that these iron ooids were formed by iron and silica associated with exhalative fluids rising from the subsurface, which deposited iron around sedimentary/skeletal particles at the seafloor or immediately below the water-sediment interface. Based on a comparison with the Indonesian ooids, Sturesson *et al*.^6^ further suggested that volcanic activity—and the consequent dissolution of associated volcanic matter—could be the source of the iron cations involved in the formation of Palaeozoic ooids from the Baltoscandian region.

**Figure 1:**
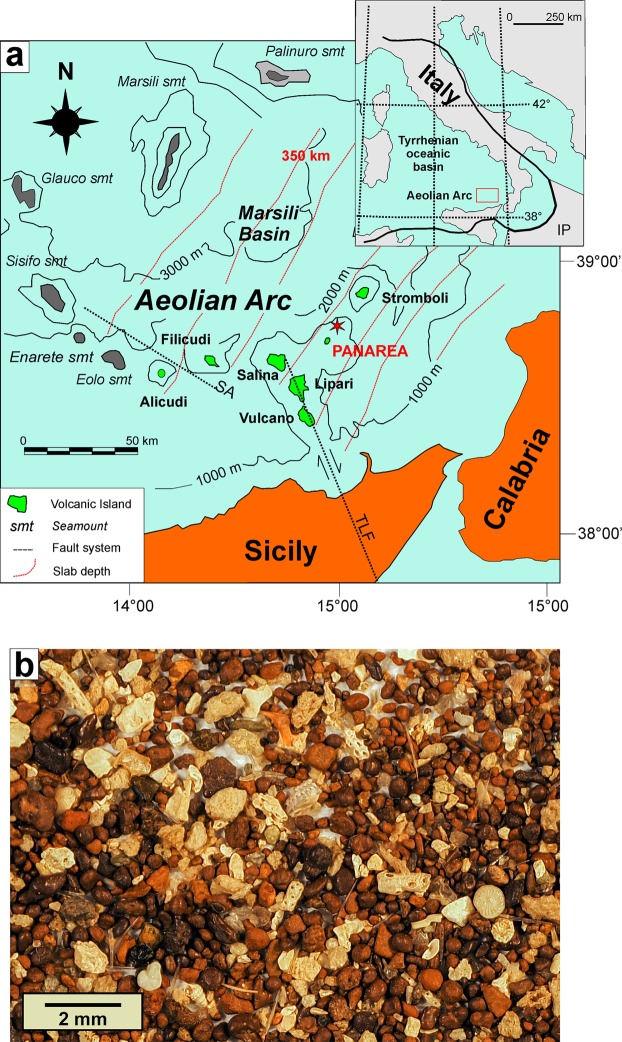
Geology of the Panarea area and material studied. (**a**) Geology of the Aeolian Islands and surrounding seamounts in the Tyrrhenian Sea (modified from^50^) and location of the study area within the Panarea Volcanic System (red star). The subduction/collision front is represented in the inset map of Italy. IP = Ionian Plate; SA = Sisifo-Alicudi fault system; smt = seamount; TLF = Tindari-Letojanni fault system. (**b**) Stereomicroscopy image of Panarea ooid sand, where a whitish biogenic component and dark rust-coloured grains (ooids) are clearly distinguishable.

This paper accounts for the still ongoing formation of iron ooids in the seabed off the island of Panarea, one of the volcanic islands of the Aeolian Arc (Tyrrhenian Sea, Italy; Fig. 1a), where shallow-water hydrothermal processes associated with intense volcanic degassing have long been documented (e.g.^18–21^). To our knowledge, this is the only place in the world where iron ooids are actively forming today. The aim of this paper is to define the processes generating the Panarea iron ooids combining the results of a variety of analytical methods. The hydrothermal constraints identified for these newly formed iron ooids will enhance the understanding on the genesis and accretion mechanisms of their fossil counterparts that are distributed at global scale throughout the Phanerozoic, and will contribute to provide a possible interpretation of analogous particles recently discovered on Mars.

## Volcanological Setting and Hydrothermal Activity of the Study Area

The large ooidal deposit discovered off the eastern coast of Panarea Island is hosted by a volcanic edifice that has evolved between 155 and 8.7 ka, following various stages of activity: between 155–149 ka the central apparatus developed then, the island of Panarea formed between 124 and 118 ka^22^. A group of islets, located to the east of the main island, is interpreted as the remnants of a crater rim^23,24^, which formed between 132.5 and 127 ky^25^. As reported by Italiano and Nuccio^18^, several hydrothermal vents around Panarea release thermal fluids at the sea bottom seeping across the sediments up to a depth of 400 m (maximum depth investigated, F. Italiano personal communication). The geochemical and microbiological features of the vented fluids (both gases and thermal waters) have been investigated since mid 1980s^18^ and studies are still ongoing (e.g.^26–28^). The collected results indicate that the vented fluids consist of thermal waters with temperatures ranging from 40 to 140 °C and CO_2_-dominated gases (e.g.^18–21^). Fluids generally emerge from open fractures and from several areas with diffuse permeation of warm waters and gases through the sandy seafloor. The composition of fluids is reported as similar to that of deep-sea hydrothermal vents^21^, and the gases consist mostly of CO_2_ plus variable concentrations of H_2_S, O_2_, CH_4_, CO, and H_2_, as well as inert gases (N_2_, Ar, He). The pH of the thermal waters ranges between 1.9 and 5.7 (see^18–20,27^). White and brown microbial mats, as well as tens of small chimneys principally composed by primary Fe-oxyhydroxides and silica, are commonly observed over the venting areas^25^. The white mat associated with the activity of sulphur cycling microbes surrounds all the areas where thermal waters discharge^23^ and largely consists of microbially mediated sulphur precipitates (e.g.^29^). The mineralized zone where the chimneys have grown are enriched in Fe and Mn, as well as metal sulphides and oxides, including galena, pyrite, marcasite, and sphalerite, as well as barite. Massive Ba-Pb-Zn-enriched sulphide deposits have recently been found to the northeast of Panarea^30^.

## Results and Discussion

### Panarea ooids

The collected material containing the ooids consist of a well sorted, unlithified sand with a light-coloured biogenic component (mainly composed of foraminifers, and minor gastropods, bryozoans, ostracodes and siliceous sponge spicules) associated to a dark, rust-coloured fraction (about 80% of the overall sample) consisting of iron ooids (Fig. 1b). About 200 ferruginous ooids were selected from the sand and hand-picked for optical and electron microscopic analyses.

**Figure 2:**
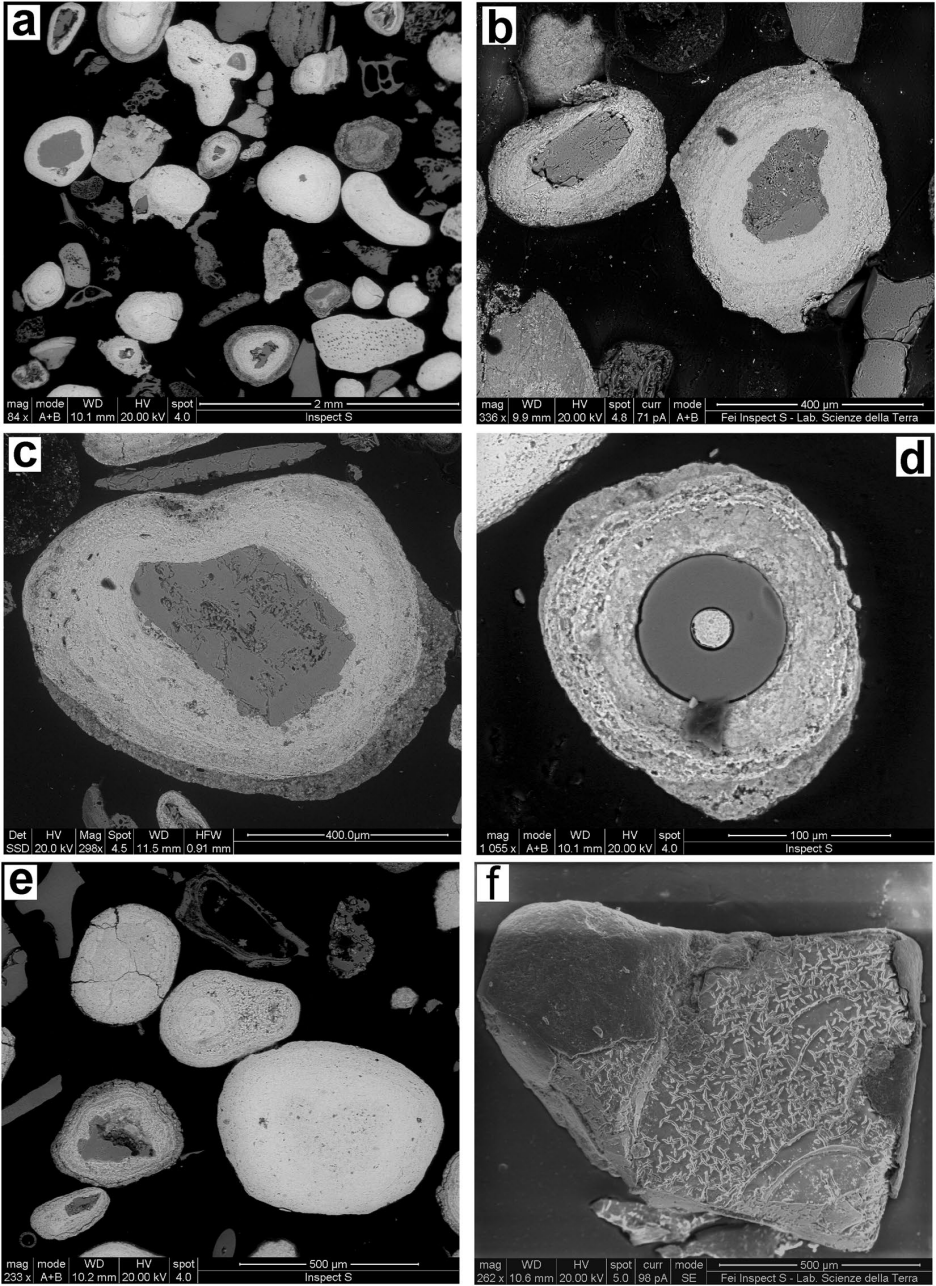
Scanning electron images of selected Panarea ooids. (**a**) Predominant ooidal morphologies. (**b**,**c**) Ooids with volcanic nuclei (pyroxene and plagioclase, respectively). (**d**) Cross section of a siliceous sponge spicula (dark grey) with goethite (light grey) filling the inner canal of the spicula and forming as well a thick external envelope made of concentric continuous laminae. (**e**) Coreless ooids. (**f**) Obsidian nucleus, partially uncovered by oxyhydroxides, revealing traces of chemical etching on the surface.

Ooids have a subspherical or elliptical overall shape (Fig. 2a), with a mean diameter ranging between 0.2 and 0.5 mm. A direct relationship between morphology and size has been reported in literature^6,10^ with larger forms (>2 mm) showing a more irregular or elliptical shape. Their formation has been explained as a faster growth of the equatorial section with respect to the slower growth of the axial one. Contrastingly, our optical and SEM-EDX observations reveal that both elliptical and spherical shapes are either associated to large (up to 8 mm) and small (<0.5 mm) size particles on which the iron-rich coatings are nucleated (Figs 2 and S1).

The excellent preservation of the ooids, with no signs of abrasion and/or fracturing, allows excluding any significant transport or reworking. Panarea ooids are characterized by the superposition of orange to dark brown 10 µm thick laminae commonly developed around a core of volcanic materials (Fig. 2b,c,f) such as pumice, glass scoria, rock fragments, or phenocrysts of volcanogenic mineral phases (Supplementary Fig. S1b–e and Supplementary Table S1). Only a few nuclei consist of siliceous sponge spicules (Figs 2d and S2a–d). Occasionally, agglomerates of two-three ooids are further coated by iron-rich laminae. Nearly one third of the ooids apparently lacks an internal nucleus (Figs 2a,e and S1a,f), and SEM images indicate for these relicts of vitreous nuclei (Fig. 2e,f), suggesting that they may have been lost due to dissolution^6,31^.

X-ray Powder Diffraction (Supplementary Fig. S3) revealed that the ooid cortex is composed of poorly crystalline goethite FeO(OH). Augite, Ca-plagioclase, and sanidine, were also detected as constituents of the internal cores, supporting a volcanic origin of the nuclei. Raman analysis (Supplementary Fig. S4) confirmed goethite as the sole iron-rich mineral phase. The lack of other iron-bearing minerals, such as hematite, and the exclusive presence of goethite suggest that no secondary (i.e., diagenetic) processes have occurred^32^.

**Figure 3:**
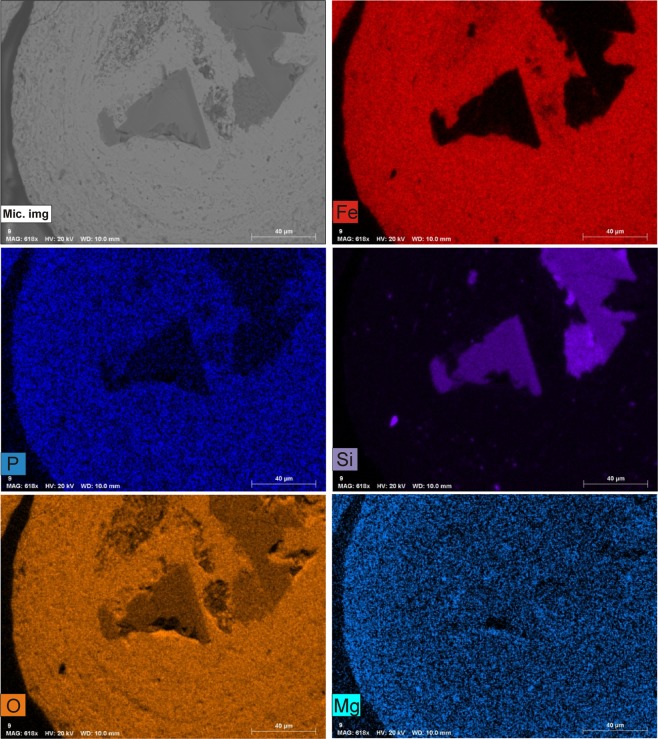
EDX elemental map distribution performed on a Panarea ooid with volcanic nucleus. The iron, phosphorus, oxygen, and magnesium EDX maps show a homogeneous distribution in the ooid cortex. Silicon is concentrated in the ooid volcanic core.

SEM-EDX chemical analyses of the ooidal cortex (Supplementary Table S2), run on approximately one hundred grains, revealed a homogeneous composition dominated by iron-rich laminae (mean FeO = 80.5 wt%) with admixed silica (mean SiO_2_ = 11.5 wt%). Manganese concentration in the iron precipitate is below the detection limit. The elemental maps of Fig. 3 confirm that the iron oxyhydroxide portion of the grains with a nucleus consisting of volcanic mineral phases is mainly composed by Fe and O and minor P, Mg and Si. XRF bulk analyses demonstrated a relatively high content of arsenic (approximately 500 ppm) and vanadium (approximately 700 ppm) among other trace elements (Supplementary Table S3). Both elements likely co-precipitated with iron in the iron hydroxide structure. All the collected chemical data are compatible with mixing phenomena involving seawater and hydrothermal fluids.

Microbial activity is well documented for the Panarea hydrothermal system and it is often described in ferruginous sediments of hydrothermal environments (e.g.^33–35^). Although a role of microbes in the ooids formation is not excluded, we did not observe any sound evidence of microbial-mediated morphologies (e.g., filaments, rods or cocci of compatible size with bacterial communities or microbial-derived morphologies, biofilm remains) in the analyzed Panarea ooids.

### Hydrothermal origin of Panarea ooids

The modern iron ooid deposit of Panarea represents a unique opportunity to constrain the origin and growth environment of both recent and fossil oolitic ironstones and of analogous Martian, iron-oxide spherules. Despite the hydrothermal precipitation of low-temperature, iron oxyhydroxide-rich red muds and crusts in the shallow-water areas around Panarea Island (e.g.^30^), no iron ooids have been found to date in other areas of the Aeolian Arc. The close association with active volcanism—together with the on-going venting of hydrothermal fluids and the limited vertical and areal confined distribution of the deposit—indicates that ooidal growth is a rapid process.

Our study suggests a scenario in which hot hydrothermal fluids (waters and CO_2_ gas emissions) seeping through seafloor sediments mix with colder seawater close to the sea water-sediment interface. These conditions favour the rapid increase in iron and silica concentration and thus their precipitation. The different colours observed in the laminae of our samples can be ascribed to variations in the Fe/Si ratio (e.g.^4^).

The primary iron oxyhydroxide and silica composition of the large chimney structures discovered in the Panarea Volcanic Complex^28^ derived from low-temperature fluids originating from the oxidation of mound sulfides^27^. We argue that the iron necessary for triggering precipitation comes from the migration of Fe^2+^ ions contained in pore fluids that rise from the deeper, reducing environment, to the water-sediment interface, where they precipitate as goethite at the boundary of the iron reduction/oxidation zone, characterized by low pH values. A similar mechanism has also been suggested for Holocene iron ooids from Indonesia^4^ and for Jurassic ooid deposits from Northern Switzerland^36^.

A further significant hydrothermal signature refers to dissolution phenomena affecting some vitreous nuclei of the studied ooids. Although dissolution of rock fragments or individual mineral phases is a time consuming process, mafic glassy shards can dissolve relatively quickly^6,31^ if driven by suitable acidic conditions and controlled by the halogen content (e.g., Cl, Br, F) of the fluids rising from the deep^37,38^. Under these conditions, it is likely that the nuclei of either obsidian fragments or glassy shards may have been partially or totally dissolved and replaced by iron oxyhydroxides. This hypothesis seems well supported by the nature of the physical and chemical parameters (i.e., low pH values, halogen contents) of the Panarea hydrothermal fluids^18,27^ and, furthermore, by the etching (dissolution) features observed on the external surface of obsidian particles when iron oxyhydroxide coverage lacks (Fig. 2f).

### A genetic model for the Panarea iron ooids

Our results support Kimberley’s^3^ claim that exhalative processes may play an important role in iron ooid formation, and these occurrences allow us to revisit the genesis of ooid microstructures found in volcanically active area of the Panarea Volcanic System. They also indicate hydrothermal emissions as the main source of iron for the precipitation of primary goethite.

Three requirements are mandatory for iron ooids formation: (i) availability of nuclei to be coated; (ii) a constant source of iron-rich fluids to facilitate the formation of mineral (goethite) coatings; and (iii) sufficient energy able to keep ooids agitated and, consequently, to ensure their growth. These three elements simultaneously occur over the Panarea hydrothermal setting.

**Figure 4:**
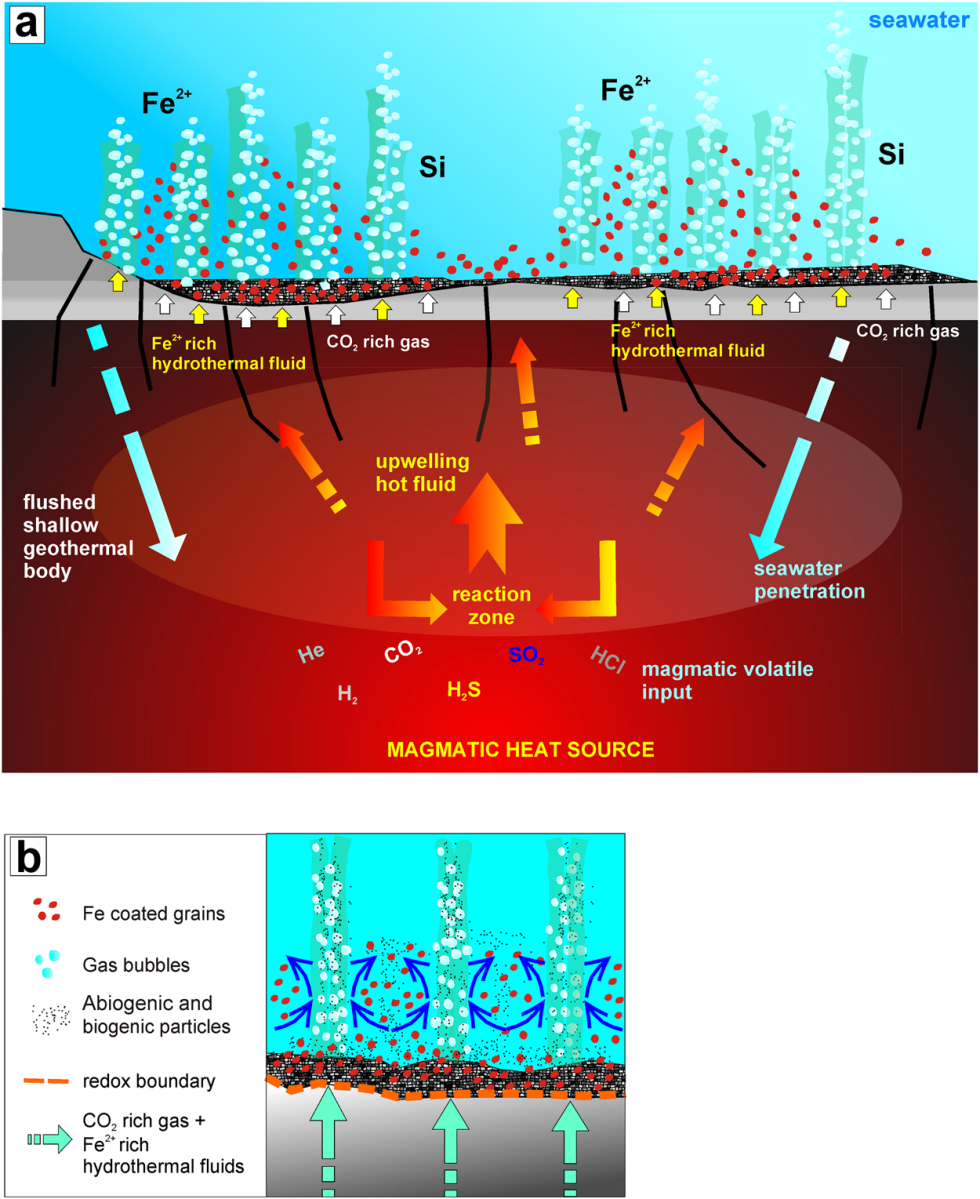
Conceptual model of ooid formation in hydrothermal environments. (**a**) Conceptual model of the submarine hydrothermal system forming ooids. The model shows (not to scale) the relationships between the CO_2_-dominated and the Fe-rich thermal fluids. (**b**) Specific mechanisms for ooid formation, where the newly formed goethite precipitates in various stages, around abiogenic (volcanic) and/or biogenic (mostly siliceous sponge spiculae) particles deposited at the seafloor.

Indeed, the genesis of Panarea ooids could be outlined by the following steps (Fig. 4):

1. Migration of Fe^2+^ ions from the deeper, reducing hydrothermal environment, towards the surface layer (sediment-water interface).
2. Chemical precipitation of the first laminae of crystalline goethite around seafloor particles of volcanic or biogenic (e.g., sponge spiculae) origin.
3. Periodic remobilization by sea waves of growing ooids (or ooid agglomerates) and consequent interruption of their growth due to incoming seabed currents and degassing of CO_2_-dominated fluids.
4. Resumption of chemical precipitation and formation of new, poorly ordered, laminae of goethite and further development of ooid coatings.
5. Steps 1 through 4 occur repeatedly until the ooids are buried in the sedimentary record.

This model - built on physical evidences that can be observed almost in real time - provides unique insight about the youngest ooid deposit known in the world, unlithified, non-reworked and unaffected by diagenetic processes, and it sheds light on the formation of the fossil deposits of iron ooids derived from shallow-water volcanism and related hydrothermal activity.

Our model could also help in interpreting the origin of similar iron spherules discovered in a number of Martian sites and, in particular, at Meridiani Planum (Martian “blueberries”), by NASA’s Mars Exploration Rover (MER) rovers^9,16,39–42^. These Martian iron spherules show morphological/dimensional (spheroidal, 0.5–8 mm) and chemical (iron oxides) analogies with our samples from Panarea.

With specific reference to the mineralogy of iron oxides, as also discussed by Glotch *et al*.^43^, goethite concretions may have been initially generated on Mars by precipitation from aqueous solutions and subsequently transformed into hematite by temperature-induced dehydroxilation (e.g.^44,45^).

Various terrestrial concretions from several environments, in search of rock formations that resemble those with the blueberries on Mars, have been proposed as Martian analogues and several formation mechanisms were suggested. The most recent model^46^ (Yoshida *et al*., 2018) discusses analogies between the iron concretions in Meridiani Planum and those present in the Jurassic Navajo Sandstone of southern Utah (USA). Based on this model, the Martian hematite spherules, as the blueberry, possibly formed by interaction between preexisting calcite spherules and acidic sulphate water that infiltrated early in Martian history.

In our opinion, the hydrothermal model proposed for the Panarea ooids better fits with the geological context and the mineralogy of the Martian surface. We propose that Martian iron spherules are homologous to the Panarea ooids, reworked and not-diagenetic, and formed during a Martian geological/volcanic phase characterized by the emission of Fe-rich hydrothermal fluids associated to CO_2_ gas (e.g.^47^). These conditions appear necessary to promote the spherical/elliptical growth of the grains and the precipitation of iron oxide.

The Martian rocks embedding the iron spherules at the Meridiani Planum include clay mineral compositions (such as in the Burns formation^48^) possibly originated from the hydrothermal alteration of volcanic rocks, formed under low pH conditions^49^. This hypothesis is further constrained by the Fe-rich and the volcanic mineral compositions detected by MER rovers on the Martian regolith, such as olivine, pyroxene, magnetite, and phyllosilicates. Also the high values of SO_3_ and jarosite mineral seems to strengthen the hypothesis of a hydrothermal origin characterized by acidic conditions.

## Material and Methods

### Sample collection and processing

An unlithified, soft sediment was sampled off the coast of Panarea Island during a research cruise carried out by ISPRA (Italian Institute for Environmental Protection and Research) and INGV on board of the research vessel Astrea. The sediment was collected at a depth of 80 m (38°40,429; 15°07,651E) NE of Basiluzzo islet. Here, venting of hydrothermal fluids extends along the slope of the escarpment toward the island of Stromboli, 80 to 400 metres below sea level, and regulated by the NE-SW and NW-SE fault system. The sampled sand contains a whitish biogenic component and dark, rust-coloured grains (ooids).

In the laboratory, the ferruginous ooidal fraction was manually separated from the biogenic material under optical microscope. A further detailed observation of the ooids was made by means of optical and electron microscopy to evidence the peculiar shape features. Powder samples and epoxide resin polished thin-sections were prepared and analyzed by X-ray Powder Diffraction (XRPD), Environmental Scanning Electron Microscopy (SEM-EDX), and Raman spectroscopy, to gain better insight into their structures, morphologies, mineralogy, and composition. To define the major and trace element composition of the ooid sand, powder pellets were produced and analyzed by wavelength dispersive X-ray Fluorescence (WDXRF). Details of each of these methods are as follows.

### Scanning electron microscopy

Spot analyses on polished sections of the iron ooids were carried out by a SEM-FEI Inspect-S equipped with an Oxford INCA PentaFETx3 EDS spectrometer and a Si(Li) detector equipped with an ultra-thin-window ATW2 (MIFT Department of the Messina University). Measurements were performed using a resolution of 137 eV at 5.9 keV. Data were acquired under environmental conditions, working at a distance of 10 mm, an acceleration voltage of 20 kV, a counting time of 60 s, and a counting rate of approximately 3,000 cps, with a dead time below 30%. The obtained semi-quantitative data were processed by INCA Energy software. This software uses the XPP matrix correction scheme developed by Pouchou and Pichoir (1990). Isolated ooids were mounted on aluminium stubs previously covered with carbon-conductive adhesive tape. Au-coated and non-coated samples were observed using a FEI ESEM-Quanta 200, equipped with an Oxford EDX INCA 300 X-ray energy dispersive spectrometer, and using a Nova Nano SEM FEI 450, equipped with a X-EDS Bruker QUANTAX-200 detector (CIGS - Modena and Reggio Emilia University). ESEM observations were performed under high and low vacuum (low vacuum brackets 1 and 0.5 Torr) with an accelerating voltage between 5 and 25 keV for imaging and between 5 and 15 keV for elemental analyses. SEM observations were performed under high vacuum with an accelerating voltage between 15 and 25 keV for imaging and between 15 and 25 keV for elemental analyses.

### X-ray powder diffraction

X-ray Powder Diffraction was performed using a Bruker D8 ADVANCE diffractometer (MIFT Department of the Messina University) with Cu Kα radiation on a Bragg-Brentano theta-theta goniometer, equipped with a SiLi solid-state detector, Sol-X. Acquisition conditions were 40 kV and 40 mA. Scans were typically obtained from 2theta values of 2 to 80 degrees, with a step size of 0.02 degrees 2theta and a count time of 1 second. Raw diffraction scans were stripped of the Kα2 component and background corrected with a digital filter (or Fourier filter). Observed peak positions are matched against the ICDD-JCPDS database.

### X-ray fluorescence spectroscopy

Wavelength dispersive X-ray Fluorescence (WDXRF) was performed using a Bruker model S8 Tiger (CERISI Laboratory of the Messina University). The excitation source was a tube of Rhat4 kW. To avoid detector saturation, the power and current intensity were varied according to the concentration and type of element analysed. The concentrations of the major and minor elements were calculated with GEO-QUANTM software. For trace elements, the GEO-QUANTT software was adopted, as it represents a simple solution for the determination of those elements in geological materials. The method was pre-calibrated and standardized by the manufacturer. This method was validated using two standard samples, GBW07103 and GBW07406.

### Raman spectroscopy

Raman analyses were carried out using a WITec Alpha 500 RA Raman spectrometer equipped with a green laser (Nd:YAG frequency doubled, wavelength 532 nm) and a Nikon E Plan 20x or 50x objective with numerical apertures of 0.4 and 0.75, respectively, at the Centre de Biophysique Moléculaire, CNRS, France. To determine the maximum laser power to be used, spot analyses with increasing laser power were performed at the centre and at the edges of the ooids.

## Supplementary information

Supplementary material
