# Supplementary material for: Modern Iron Ooids of Hydrothermal Origin as a Proxy for Ancient Deposits
Source: Sci Rep. 2019 May 8;9:7107. doi: 10.1038/s41598-019-43181-y (PMC6506468; doi:10.1038/s41598-019-43181-y)
Supplement: Supplementary file 1 — Supplementary material [file 41598_2019_43181_MOESM1_ESM.pdf]

SUPPLEMENTARY INFORMATION

MODERN IRON OOIDS OF HYDROTHERMAL ORIGIN AS A PROXY FOR ANCIENT DEPOSITS

Marcella Di Bella[1], Giuseppe Sabatino[2], Simona Quartieri[2], Annalisa Ferretti[3], Barbara Cavalazzi[4,5], Roberto Barbieri[4], Frédéric Foucher[6], Fabio Messori[3,7],Francesco Italiano[1*]

[1]Istituto Nazionale di Geofisica e Vulcanologia (INGV), Sezione di Palermo, Via Ugo La Malfa 153, 90146 Palermo, Italy

[2]Dipartimento di Scienze Matematiche e Informatiche, Scienze Fisiche e Scienze della Terra (MIFT), Università di Messina, Viale Ferdinando Stagno d'Alcontres 31, 98166S. Agata, Messina, Italy

[3]Dipartimento di Scienze Chimiche e Geologiche (DSCG), Università di Modena e Reggio Emilia, Via Campi 103, 41125 Modena, Italy

[4]Dipartimento di Scienze Biologiche, Geologiche e Ambientali (BiGeA), Università di Bologna, Via Zamboni 67, 40126 Bologna, Italy

[5]Department of Geology, University of Johannesburg, PO Box 524 Auckland Park 2006, Johannesburg, South Africa

[6]Centre de Biophysique Moléculaire (CBM), Rue Charles Sadron, 45071 Orléans Cedex 2, France

[7]Department of Earth Sciences, University of Geneva, Rue des Maraîchers 13, 1205 Geneva, Switzerland

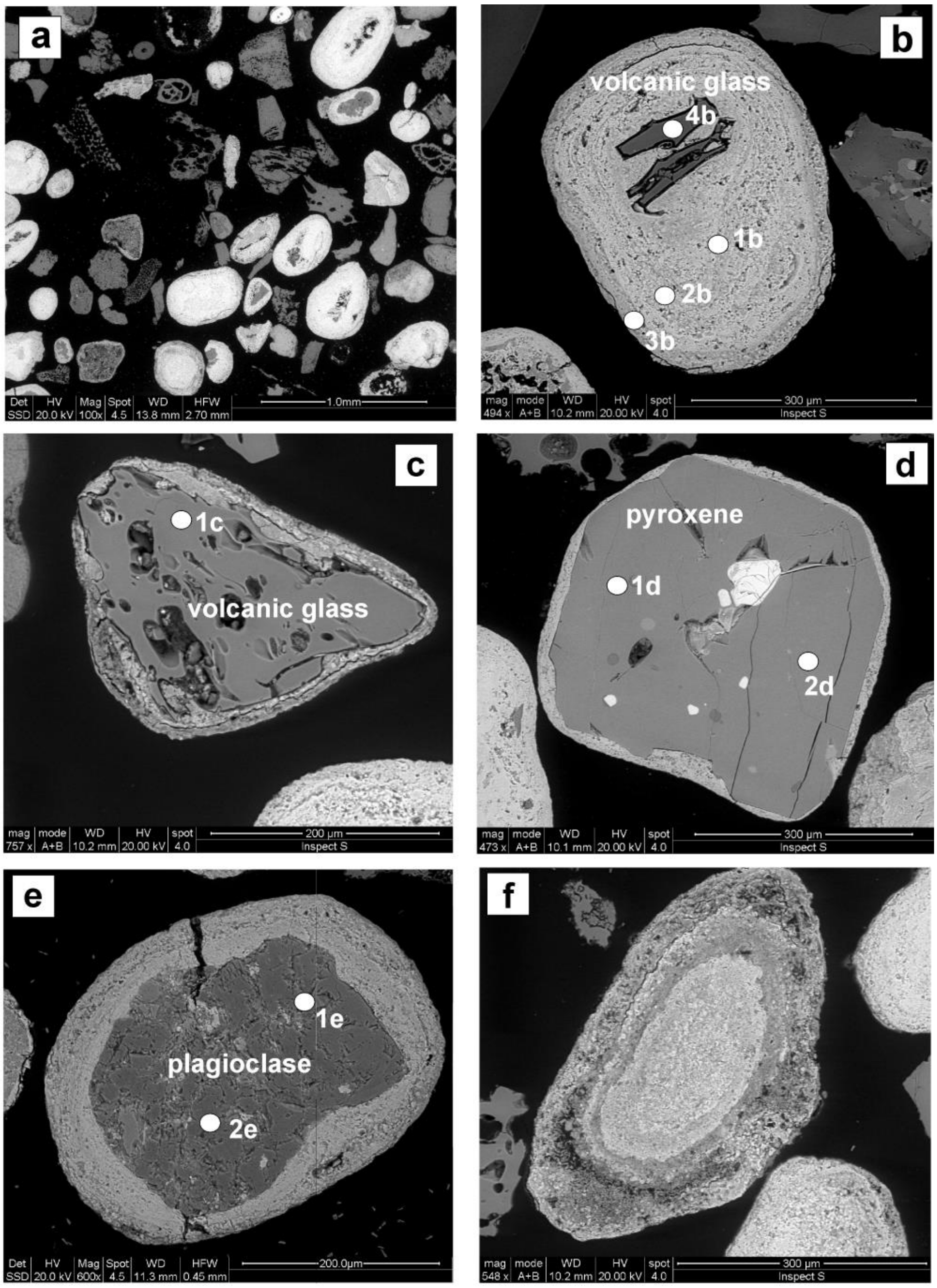

Ooid Fig. 2b	SiO_2 wt%	Al_2O_3 wt%	FeO wt%	Cl wt%
**internal - 1b**	9.97	-	81.63	-
**intermediate - 2b**	10.23	2.98	85.95	0.84
**external - 2c**	11.78	3.48	79.41	0.76

Figure S1. SEM images of some Panarea ooids, showing the internal nuclei made of fragment of volcanic phases. (**a**) General view of the main ooid morphologies in thin-sectioned material;(**b**, **c**) examples of nuclei containing fragments of volcanic glass; (**d, e**) nuclei containing pyroxene and plagioclase crystals; (**f**) example of coreless ooid. The underlying table reports SEM-EDX

compositional data of external, intermediate and internal areas of iron-oxydroxydes ooid cortex reported in figure 2**b**.

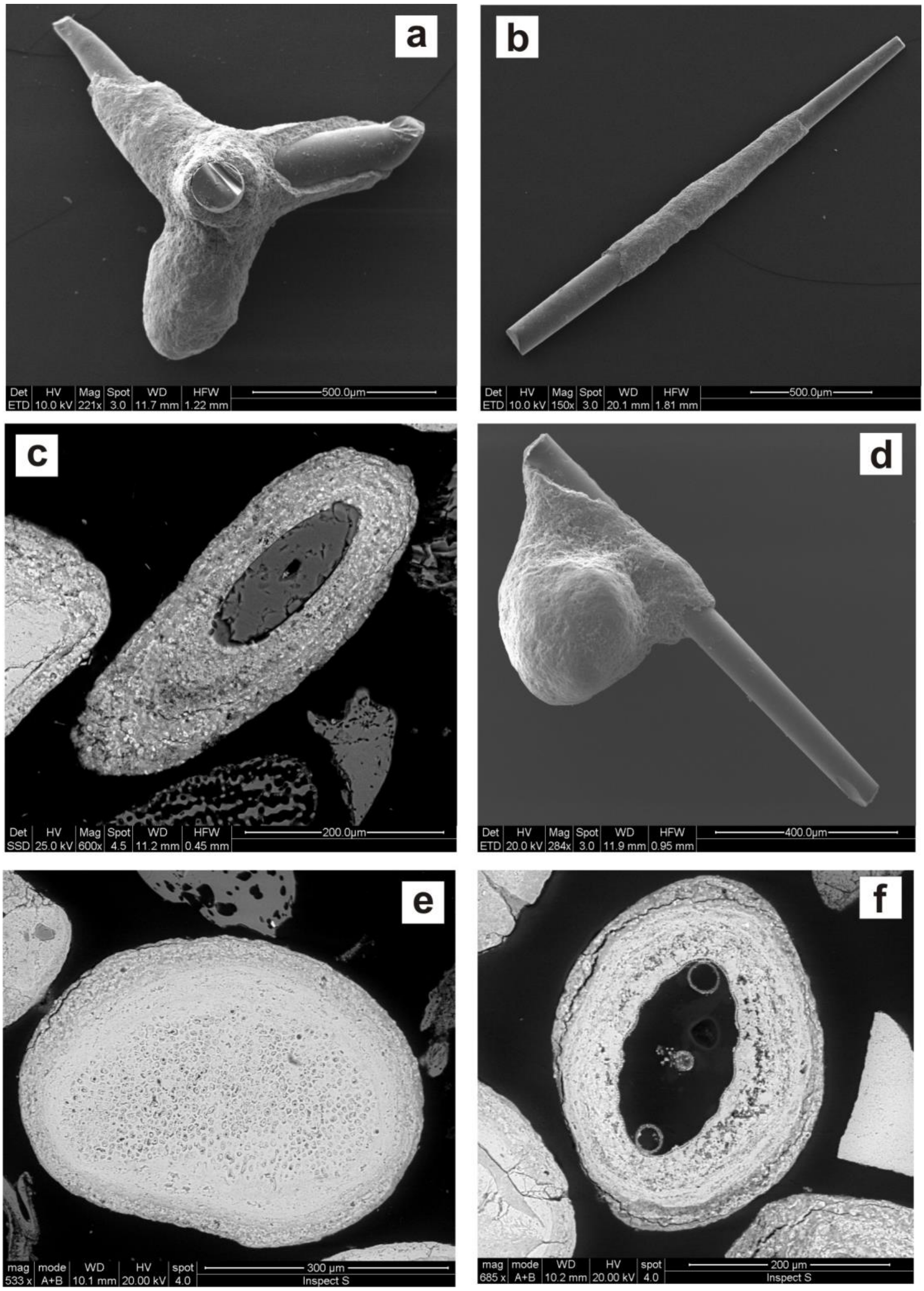

Figure S2. SEM images of selected Panarea ooids developed around biogenic particles. Nuclei are mainly represented by siliceous sponge spiculae (**a**-**d**) and more rarely by undetermined bioclasts (**e** and **f**).

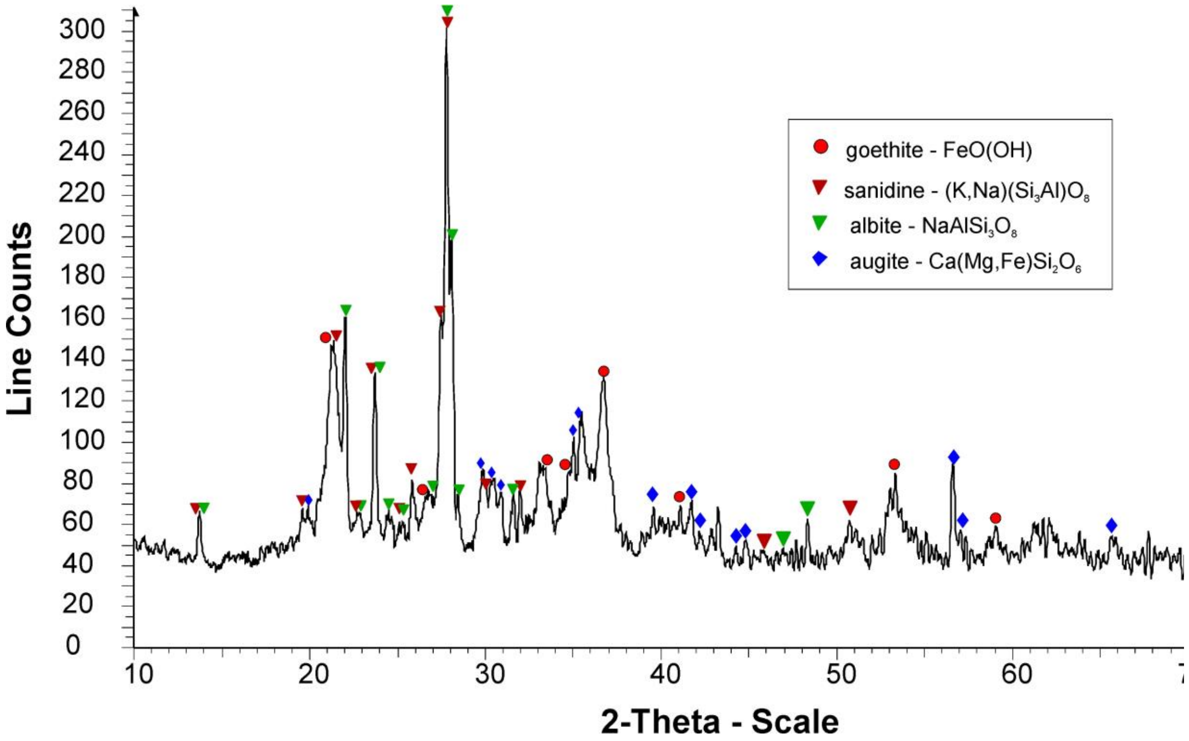

Figure S3. X-ray diffractometric pattern of Panarea ooids. The identified minerals are goethite, as the sole Fe-rich mineral, and the volcanic phases sanidine, albite and augite.

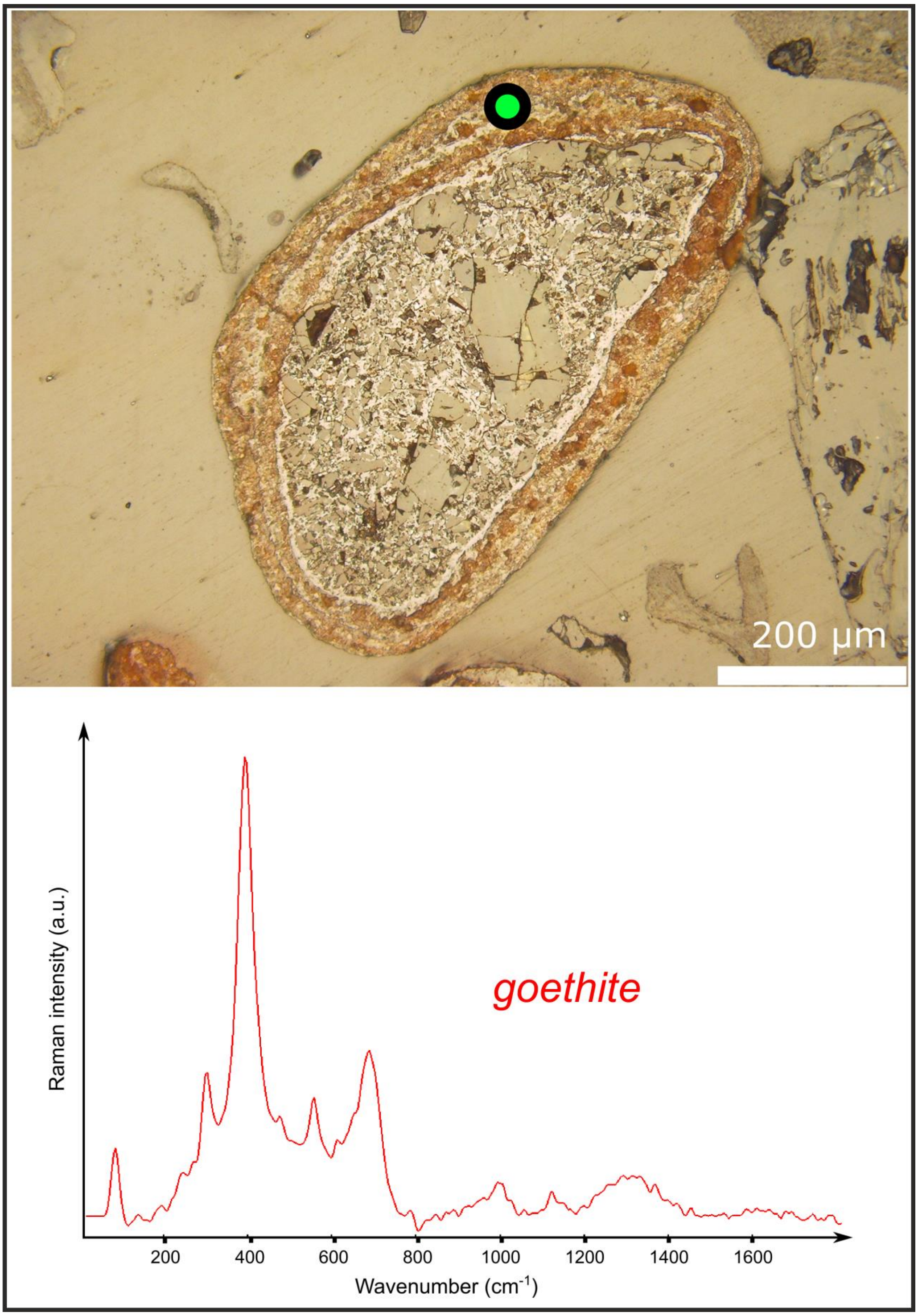

Figure S4. Optical microscopy image in reflected light of an iron ooid and associated Raman spectrum, confirming the presence of goethite in the ooid cortex. Position of the spot analysis is indicated by the green circle.

Table S1. Composition of the mineral phases identified within nuclei of the Panarea ooids shown in Figure S1 by an integration of petrographic and SEM/EDX analyses. For pyroxene and plagioclase, the calculated mineralogical formulae are also reported.

**Mineral phase oxides wt %**	**pyroxene**		**plagioclase**			**volcanic glass**		
**Point analysis**	**1d**	**2d**		**1e**	**2e**		**1c**	**4b**
**SiO_2**	50.77	52.84	**Na_2O**	5.66	5.31	**Na_2O**	4.98	3.29
**TiO_2**	0.86	0.46	**Al_2O_3**	26.49	26.86	**Al_2O_3**	12.82	11.74
**Al_2O_3**	4.29	4.69	**SiO_2**	58.37	57.42	**SiO_2**	75.32	73.25
**FeO**	13.17	8.88	**K_2O**	0.52	0.36	**K_2O**	3.79	4.46
**MgO**	13.13	16.62	**CaO**	8.88	9.00	**FeO**	3.08	7.26
**CaO**	17.79	16.52	**FeO**	0.36	0.6500	**Total**	99.99	100
**Total**	100.01	100.01	**Total**	100.28	99.60			
**Cations**			**Cations**					
**Si**	1.9031	1.9031	**Si**	2.6057	2.5823			
**Ti**	0.0242	0.0242	**Al**	1.3937	1.4237			
**Al**	0.1895	0.1895	**Fe^{2+}**	0.0134	0.0244			
**Fe^{2+}**	0.4129	0.4129	**Ca**	0.4247	0.4337			
**Mg**	0.7337	0.7337	**Na**	0.4899	0.4630			
**Ca**	0.7145	0.7145	**K**	0.0296	0.0207			
**Total**	3.9779	3.9779	**Total**	4.9572	4.9477			
**End Members %**			**End Members %**					
**Wo**	38.39	38.39	**Ab**	51.88	50.47			
**En**	39.42	39.42	**An**	44.98	47.27			
**Fs**	22.18	22.18	**Or**	3.14	2.25			

Table S2. Representative SEM-EDX major element composition of selected ooid samples.

SAMPLES	SiO_2	Al_2O_3	FeO	MgO wt%	CaO	P_2O_5	Cl
**Ooid1**	14.07	4.59	76.79	1.71	-	2.84	-
sigma	0.23	0.19	0.51	0.2	-	0.17	-
**Ooid2**	9.82	2.91	84.5	-	-	1.95	0.75
sigma	0.37	0.33	0.92	-	-	0.3	0.23
**Ooid3**	11.78	3.48	79.41	1.71	-	2.76	0.76
sigma	0.31	0.25	0.74	0.28	-	0.23	0.19
**Ooid4**	10.01	6.73	77.5	-	-	3.88	1.65
sigma	0.7	0.65	1.6	-	-	0.57	0.5
**Ooid5**	11.08	1.98	82.55	1.76	0.59	2.04	-
sigma	0.14	0.11	0.35	0.14	0.07	0.1	-
**Ooid 6**	14	3.85	77.23	1.67	0.9	1.88	-
sigma	0.17	0.13	0.38	0.14	0.09	0.12	-
**Ooid7**	10.34	2.85	83.01	1.77	-	1.62	0.37
sigma	0.45	0.39	1.14	0.46	-	0.35	0.29
**Ooid8**	12.57	4.16	78.18	1.28	-	3.15	0.58
sigma	0.32	0.27	0.75	0.28	-	0.24	0.19
**Ooid9**	9.56	1.66	87.63	1.14	-	-	-
sigma	0.38	0.33	0.93	0.37	-	-	-
**Ooid10**	12.44	4.85	79.01	1.32	-	1.8	0.52
sigma	0.44	0.33	1.12	0.46	-	0.37	0.24
**Mean**	11.57	3.71	80.58	1.55	0.75	2.44	0.77

Sigma: analytical error; -: below detection limits.

Table S3. X-ray fluorescence data of minor and some trace elements for four representative samples (powder pellets) of the Panarea ooids.

		**sample 1**		**sample 2**		**sample 3**		**sample 4**	
**Elements**	D.L.	**ppm**	s.e.	**ppm**	s.e.	**ppm**	s.e.	**ppm**	s.e.
**V**	1.4	717	0.25	1030	0.2	951	0.33	795	0.21
**Cr**	3.3	39	4.1	33	4.12	25	3.95	45	3.80
**Ni**	1.5	26	4.15	61	2.63	55	4.26	35	3.90
**Cu**	4.7	28	6.31	45	6.36	42	5.86	34	5.93
**Zn**	3.8	100	1.44	160	1.41	127	1.45	153	1.10
**As**	2.9	496	0.41	728	0.38	682	0.48	535	0.56
**Rb**	0.9	76	0.89	34	2.38	33	1.61	76	0.88
**Sr**	0.9	384	0.23	114	0.76	81	0.69	407	0.28
**Y**	6.5	33	1.25	38	1.71	34	1.16	34	1.59
**Zr**	4.9	78	0.51	39	1.41	35	1.28	82	0.44
**Nb**	0.6	12	3.06	6	7.3	11	2.89	13	2.19
**Mo**	1	40	1.49	110	0.71	108	0.88	46	1.05
**Ba**	5	126	2.29	90	3.39	88	3.23	201	2.01
**Ce**	4	46	7.42	31	11.5	24	10.68	55	7.37
**Pb**	-	69	1.12	45	2.33	40	1.32	74	1.01
**Th**	1.5	20	4.43	13	12.4	11	11.53	20	3.76
**U**	1.5	-	-	10	12.9	13	11.19	-	-

s.e.: standard error; ppm: parts per million; D.L.: detection limit; - : below detection limits
